# An inverse association between tea consumption and colorectal cancer risk

**DOI:** 10.18632/oncotarget.16959

**Published:** 2017-04-08

**Authors:** Yuetong Chen, Yuan Wu, Mulong Du, Haiyan Chu, Lingjun Zhu, Na Tong, Zhengdong Zhang, Meilin Wang, Dongying Gu, Jinfei Chen

**Affiliations:** ^1^ Department of Oncology, Nanjing First Hospital, Nanjing Medical University, Nanjing, China; ^2^ Department of Environmental Genomics, Jiangsu Key Laboratory of Cancer Biomarkers, Prevention and Treatment, Collaborative Innovation Center for Cancer Personalized Medicine, Nanjing Medical University, Nanjing, China; ^3^ Department of Medical Oncology, Jiangsu Cancer Hospital, Nanjing Medical University, Nanjing, China; ^4^ Department of Genetic Toxicology, The Key Laboratory of Modern Toxicology of Ministry of Education, School of Public Health, Nanjing Medical University, Nanjing, China; ^5^ Department of Biostatistics, School of Public Health, Nanjing Medical University, Nanjing, China; ^6^ Department of Oncology, The First Affiliated Hospital of Nanjing Medical University, Nanjing, China

**Keywords:** colorectal cancer, inverse association, tea consumption

## Abstract

It is well known that the tea extracts, mainly polyphenols as chemo-preventive elements, could act as cancer progression blockers. Although the association between tea consumption and colorectal cancer risk has been widely investigated, the results still remain inconsistent. We conducted a dose-response meta-analysis to evaluate their relationships by enrolling qualified 29 literatures. The summary odds ratio (OR) of colorectal cancer for the highest *vs*. lowest tea consumption was 0.93 with 0.87–1.00 of 95% confidence intervals (CIs) among all studies with modest heterogeneity (*P* = 0.001, *I^2^* = 43.4%). Stratified analysis revealed that tea, especially green tea, had a protective effect among female and rectal cancer patients. Particularly, the dose-response analysis showed that there was a significant inverse association between an increment of 1 cup/day of tea consumption and colorectal cancer risk in the subgroup of the green tea drinking (OR = 0.98, 95% CI = 0.96–1.01, *P*_nonlinear_ = 0.003) and female (OR = 0.68, 95% CI = 0.56-0.81, *P*_nonlinear_ < 0.001). Our findings indicate that tea consumption has an inverse impact on colorectal cancer risk, which may have significant public health implications in the prevention of colorectal cancer and further similar researches.

## INTRODUCTION

Colorectal cancer (CRC) is one of the most common malignances occurred in the digestive system. Although the diagnosis and treatment of CRC have been improved in the last decades, the incidence of CRC presents an upward trend [1], more seriously the 5-year survival rate of patients diagnosed as metastatic CRC is < 15% [2]. CRC has become the third most common cancer for both the male and female [2]. As colorectal cancer is still a challenging global health problem, urgent primary prevention strategies are warranted.

Emerging evidences have demonstrated that dietary factors, including tea intake, could contribute to the prevention of CRC [3]. Many studies focused on the functions of tea in the development and progress of CRC, because tea is a kind of common worldwide beverage and known as a chemo-preventive actor for various diseases [4, 5]. Epidemiological studies have indicated that epigallocatechin-3-gallate (EGCG), the most luxuriant and vigorous polyphenol in green tea, could modulate the signal transduction and metabolic pathways. It can cause cellular behavior changes, including apoptosis, proliferation and angiogenesis [3, 6]. However, the results were still controversial. Several studies even suggested that tea containing mutagenic and genotoxic compounds could raise the risk of CRC [7].

In the last decades, many epidemiologic studies have evaluated the connection between tea consumption and CRC morbidity and mortality. Nevertheless, the findings have not reached a consensus currently. Some of the inconsistencies were contributed by the discrepancy in tea-drinking habits, types of tea source and the amount of consumption [8]. Besides, failure of the modulation of potential confounding factors and inadequate assessment of tea consumption may affect the conclusion [8].

In this study, we performed a dose-response meta-analysis of cohort studies and case-control studies with the aims: (a) to estimate the association between tea consumption and CRC risk; (b) to examine the relevance degree of tea consumption and CRC risk according to study designs, study populations, genders, cancer subtypes, tea source and different degrees of adjustment for confounding factors; and (c) to evaluated the dose-response patterns of tea consumption on the risk of CRC.

## RESULTS

### Search results, study characteristics and quality assessment

**Figure 1:**
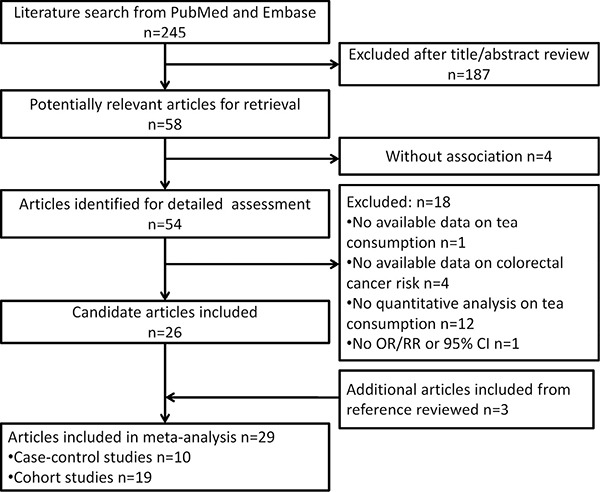
Flowchart of study selection

Figure 1 presents the study selection process. Briefly, a total of 245 literatures were initially screened from PubMed and Embase, of which 54 were identified for detailed evaluation with full-text retrieval. Subsequently, 18 of 54 articles were excluded for the following reasons: no available data on tea consumption (*n* = 1) or CRC risk (*n* = 4), no quantitative analysis on tea consumption (*n* = 12) and no odds ratios/relative risks (ORs/RRs) or 95% confidence internals (CIs) (*n* = 1). Three additional articles were included from the further reference review [9–11]. Totally, 29 articles [4, 9–36], including 12 case-control studies and 17 cohort studies with a total of 1,642,007 subjects (46,467 in case-control studies and 1,595,540 in cohort studies), were recruited for meta-analysis (Supplementary Table 1). The detailed quality scores of all studies are summarized in Supplementary Tables 2 and 3, respectively. The quality scores ranged from 5 to 8, and the median score was 6.5 for case-control studies and 6.7 for cohort studies.

### Tea consumption and colorectal cancer risk

**Figure 2:**
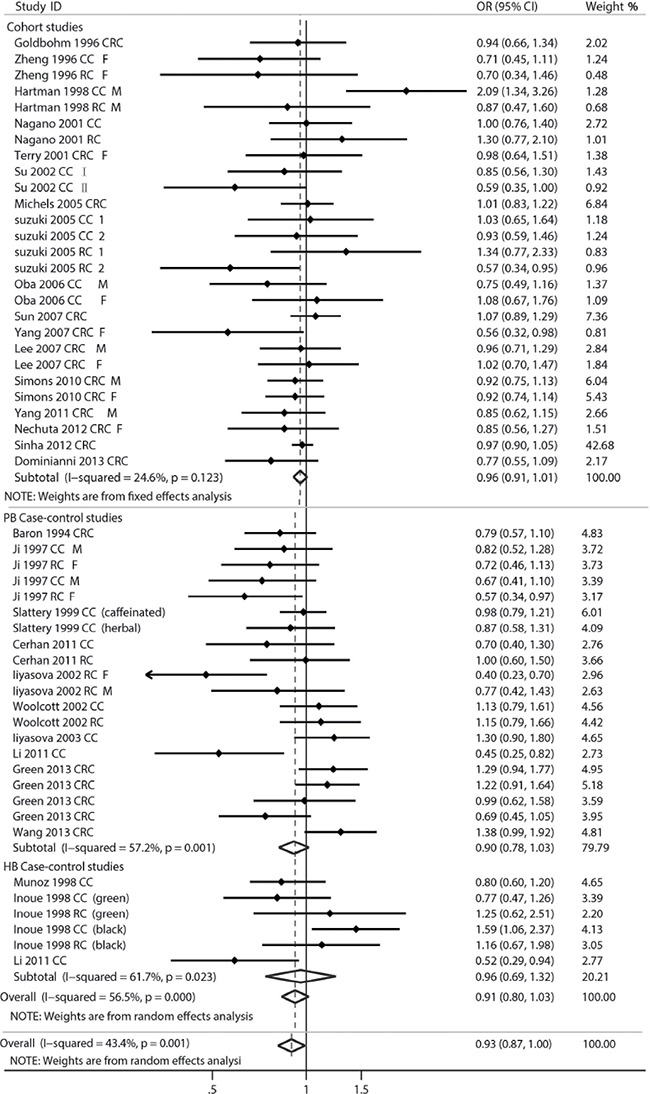
Forest plot of tea consumption and colorectal cancer risk HCC, hospital-based case-control study; PCC, population-based case-control study; CRC, colorectal cancer; CC, colon cancer; RC, rectal cancer; M, male; F, female.

The multivariable-adjusted ORs/RRs for each study were extracted and the combined ORs for the highest *vs*. the lowest classes of tea consumption are shown in Figure 2. Overall, the pooled OR for highest compared with the lowest tea consumption was 0.93 (95% CI = 0.87–1.00) with modest heterogeneity (*P* = 0.001, *I*^2^ = 43.4%), showing inversely relationship between tea drinking and CRC risk.

### Subgroup analysis

Subsequently, we performed stratified analysis to evaluate the relationship between tea consumption and CRC risk (Table 1). When we stratified for study design in this meta-analysis, significant inverse associations were observed in green tea studies (OR = 0.87, 95% CI = 0.76–0.98), especially in female (OR = 0.86, 95% CI = 0.78–0.94). It is suggested that women who prefer drinking green tea have more chance of avoiding colorectal cancer. Stratification across CRC locations demonstrated the statistical significance in the rectal cancer group (OR = 0.91, 95% CI = 0.85–0.99). Besides, we also tried to analysis the difference between design formations and geographic areas, but no significant associations were found.

**Table 1:** Stratified analyses of tea consumption and colorectal cancer risk

Group	Studies (*n*)	OR (95% CI)	Heterogeneity test		
			*χ2*	***P***	*I^2^*(%)
Total	29^a^	0.93 (0.87–1.00)	91.94	0.001	43.4
Design					
Cohort	17	0.96 (0.91–1.01)	34.50	0.123	24.6
Case-control	12	0.91 (0.80–1.03)	57.43	0.000	56.5
Population based	10	0.90 (0.78–1.03)	26.64	0.001	57.2
Hospital based	3	0.96 (0.69–1.32)	12.06	0.023	61.7
Geographic area					
Asia	12	0.91 (0.81–1.02)	44.89	0.006	46.5
Europe	5	0.99 (0.82–1.20)	13.55	0.035	55.7
America	11	0.95 (0.89–1.00)	24.91	0.071	35.8
Australia	1	1.09 (0.91–1.30)	6.29	0.098	52.3
Tea source					
Green tea	10	0.87 (0.76–0.98)	33.66	0.002	43.6
Any tea	22	0.97 (0.90–1.05)	60.56	0.002	45.5
Sex					
Male	13	0.98 (0.85–1.12)	35.40	0.008	49.2
Female	15	0.86 (0.78–0.94)	18.07	0.349	8.5
Location					
Colon	22	0.92 (0.84–1.01)	51.83	0.008	42.1
Rectal	19	0.91 (0.85–0.99)	36.73	0.051	31.3
Publication year					
Before 2000	8	0.91 (0.79–1.06)	32.03	0.010	50.0
2000 or later	21	0.94 (0.87–1.01)	59.28	0.006	41.0
Quality score					
< 7 stars	13	0.89 (0.81–0.99)	47.10	0.007	44.8
≥ 7 stars	16	0.97 (0.89–1.06)	41.61	0.020	39.9

^a^A total of 29 literatures were enrolled in our study, among which may include more than one study for meta-analysis.

### Dose-response meta-analysis

**Figure 3:**
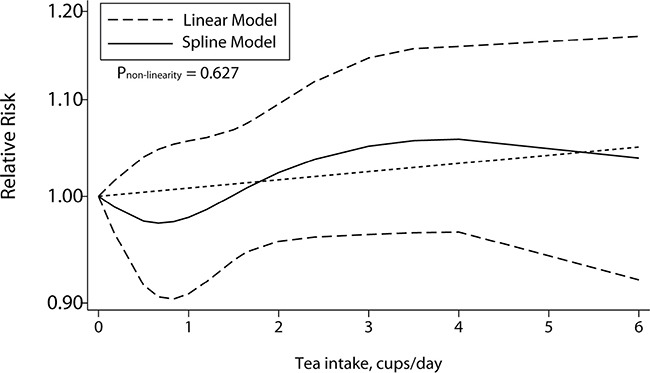
Dose-response association between tea consumption and colorectal cancer risk

A total of 10 studies including 5 cohort studies and 5 case-control studies, offering the number of cases and controls or person-years and ORs/RRs with 95% CI for at least 3 quantitative exposure levels, were enrolled for the dose-response meta-analysis. No significant evidence supported the association of an increase in tea consumption of 1 cup/day and CRC risk (OR = 1.01, 95% CI = 0.99–1.03, Table 2) with no nonlinear correlation (*P* = 0.627, Figure 3). However, the results for stratified analysis showed a significant inversion between the increment of 1 cup/day tea intake and the risk of CRC. As shown in Table 2 stratified by geographic area, tea source and sex, significant nonlinear relationship with an increment of 1 cup/day was identified in the subgroup of green tea source (OR = 0.98, 95% CI = 0.96–1.01, *P*_nonlinear_ = 0.003) and female (OR = 0.68, 95% CI = 0.56–0.81, *P*_nonlinear_ < 0.001) without heterogeneity.

**Table 2:** Subgroup analysis for risk estimates of increment of 1 cup/day of tea consumption with colorectal cancer risk

	OR (95% CI)	***P***_heterogeneity_	***P***_nonlinear_
Total	1.01 (0.99–1.03)	0.028	0.627
Geographic area			
Asia	0.98 (0.96–1.01)	0.081	0.063
Tea source			
Green tea	0.98 (0.96–1.01)	0.260	0.003
Sex			
Female	0.68 (0.56–0.81)	0.406	< 0.001

### Sensitivity analysis

We designed a sensitivity analysis to investigate potential sources of heterogeneity. We excluded 9 studies without adjusted for smoking or passive smoking, and analyzed the rest studies. The result yielded a pooled OR = 0.94 (95% CI = 0.87–1.00) with marginal heterogeneity (*P* = 0.021, *I*^2^ = 34.0%). Besides, the exclusion of another 9 studies not controlled for alcohol drinking revealed an OR of 0.87 (95% CI = 0.74–1.02, *P* = 0.001, *I*^2^ = 58.2%). Furthermore, exclusion of any single study presented the similar overall results, with a range from 0.92 (95% CI = 0.86–0.98) to 0.95 (95% CI = 0.89–1.01).

### Meta-regression and publication bias

The factors including study design, region, tea sources, gender and publish year were taken into consideration for meta-regression analysis, but none of them showed significant source of heterogeneity (Supplementary Table 4). The estimated between-study variance (τ^2^) was increased from 0.019 to 0.030 when we analyzed the influence of those five factors in the meta-regression. As shown in Supplementary Figure 1, there was no significant publication bias in the studies on highest *vs*. lowest level of tea consumption and CRC risk with *P* = 0.070 of Egger test.

## DISCUSSION

In recent years, the association of tea consumption and CRC development has been widely investigated, but the findings remain controversial. A cohort study performed in the United States suggested that there seemed no inverse association between tea consumption and CRC [36]. In contrast, another study conducted in Shanghai showed that regular tea consumption may reduce the risk of CRC [34]. In this study, we performed a meta-analysis to comprehensively evaluate the association between tea consumption and colorectal cancer risk, mainly based on 12 case-control and 17 cohort studies. We found that high tea consumption was significantly associated with reduced of CRC risk, especially in the subgroup of female, green tea source and rectal cancer. Moreover, the dose-response meta-analysis also verified that there was a significant inversion between an increment of tea consumption of 1 cup/day and colorectal cancer risk in the subgroup of green tea and the female population.

It is well known that CRC, as one of the major causes of mortality of death in the world, is mainly caused by genetic and environmental factors. Immigration epidemiological studies have reported that after the residents in low-incidence countries moved to high-incidence regions, the morbidity of colorectal cancer increased rapidly [37], which primarily attributed to the changes of dietary habit and lifestyle [38]. Tea, as one of the most popular beverages consumed worldwide, has been widely investigated the effects on human health.

Many studies, including animal and cell experiments, have revealed the anti-cancer effect of tea extracts on cancer development and progress [39, 40]. Recently, Chen et al. has indicated that tea polyphenols, one of tea extracts, could induce cancer cell apoptosis by suppressing Survivin expression [41]. Besides, EGCG, a major component of green tea, has been reported to perform multiple protective effects on human disease, including anticancer, neuroprotective and DNA-protective effects. The ingredient may be involved in colorectal cancer cell proliferation and apoptosis by increasing caspase-3/-8/-9 activity and protect normal cells against DNA-damage [42]. However, in contrast to laboratory evidence for the cancer preventive functions of tea constituents, human epidemiological studies are not strongly consistent in demonstrating the cancer preventive effect of tea consumption. Recently, Yang et al. has reviewed cohort studies and reported no relationship between tea consumption and endometrial cancer risk [43], as well as in breast cancer [44], gastric cancer [45], but not in lung cancer [46].

The latest meta-analysis on the relationship between tea consumption and CRC risk was reported by Wang et al. [47], which only enrolled 5 green tea consumption cohort studies [24, 28, 31, 32, 34]. Their findings showed that green tea could present an inverse association with incidence of colorectal and colon cancer only in Shanghai population, and even a higher CRC risk in Singapore men. Nevertheless, they did not observe an association of the increment of 1 cup/day in green tea consumption with colorectal cancer incidence. They excluded the case-control studies and only recruited 5 literatures for meta-analysis, limiting the explanation of their conclusion as for the small sample size of studies.

In the present study, we enrolled all available case-control studies and cohort studies, which involved CRC risk and tea consumption. In our study, only 46,467 participants were included in case–control studies, whereas 1,595,540 subjects in cohort studies. We know that the conclusion from cohort studies is considered preferable to that from case–control studies owning to their scientific and strict evidence. Additionally, more protective effects of tea consumption were identified in the subgroup of the Asians, green tea drinking and female. In the further dose-response analysis, we identified a significant association without heterogeneity between an increment of 1 cup/day of tea consumption and colorectal cancer risk in the subgroup of the female population. These findings were mainly on account of the increasing tea consumption and healthier lifestyle in the female worldwide [48]. Considering that all ORs and 95% CIs are roughly 1, it may draw a conclusion that tea consumption could be a slightly primary prevention strategy for CRC because different study designs can be affected by individual genetic variations, environment factors and dietary habits, to name a few. Besides, the differences between ORs of articles evaluated by < 7 stars and ≥ 7 stars may derive from improvement and enhancement of patients data bases, study designs and analysis methods.

A strength of this study was that we enrolled all relevant studies (*n* = 29), whose data was substantial and significantly increased the statistical power of the analysis. Besides, a significant dose-response association between tea consumption level and CRC risk further strengthened the association. However, our study still has several limitations. First, heterogeneity cannot be fully eliminated, neither in overall nor in subgroups analysis. This might be for the different range of the highest and the lowest categories for tea consumption among enrolled studies. Second, the methods of calculating the tea consumption were different among all studies. Though we converted the amount of tea consumption to cups/day before performing the analysis, there still might remain some bias, resulting in the deviation of risk estimate values and confounding factors. Third, several studies only provided one or two reports while some included almost 6 reports. The bias of the quality and quantity of cases in included reports could influence the final results. Fourth, most of the included studies only displayed the results without providing the detailed calculation methods or the raw data (i.e., the person-years at different levels of tea consumption) which may cause bias in results of the dose-response analysis. Fifth, the analysis methods and software and the limitation of our knowledge may account for the statistical bias. Above all, these results should be considered with caution.

In summary, this meta-analysis indicates that tea consumption could be a primary protective lifestyle to CRC. Further well-designed large prospective studies and randomized clinical researches are warranted to confirm these relationships and to establish the exact dose-response relationship. Moreover, mechanisms in the field are needed to be studied to realize precise primary protection of CRC.

## MATERIALS AND METHODS

### Literature search strategy

The literature search was conducted in PubMed and Embase up to June 1, 2016 by using the key words as follows: “colorectal” OR “colon” OR “rectal” AND “cancer” OR “carcinoma” OR ”neoplasm” OR “tumor” AND “tea” OR “drinking” OR “beverages” OR “diet”. All articles were limited to English language. The reference lists were also checked for study inclusion/exclusion. Two independent investigators (Y.C. and M.D.) searched and retrieved potential studies. Our research was designed, conducted and reported according to the standards of quality for reporting mate-analyses.

### Study selection

Studies were selected for meta-analysis have to achieve the following criteria: (a) designed by case-control or cohort study; (b) evaluated the associations between tea consumption and CRC risk; (c) all CRC cases were either histopathologically or cytologically confirmed; (d) provided the quantity of CRC cases and controls or person-years; (e) supplied relative risks (RRs) or odds ratios (ORs) with corresponding 95% confidence intervals (CIs), especially for highest *vs*. non/lowest level of tea consumption. For those on which a dose-response analysis can be conducted, we resolved all disagreements by consensus.

### Data extraction and quality assessment

The data extraction was conducted by two reviewers (Y.C. and M.D.) referring to the MOOSE (meta-analysis of observation studies in epidemiology) guidelines [49], and discrepancies could be discussed with the third reviewers (Y. W. and H.C.) to reach a consensus, which contributed to the data accuracy. For each included study, the required data was extracted as follows: first author, publication year, country, study design, cancer site, follow-up period, sample size, gender, age, type of tea, amount of tea consumption, adjusted RRs or ORs for highest *vs*. lowest intake and 95% CIs and adjustment confounding variables. The evaluation system based on the Newcastle-Ottawa Scale was used to evaluate the study quality [50] by two investigators independently from 3 parts: the screened study population, the comparability of subjects, and confirmation of exposure for case-control or cohort studies. The full score is 10 stars, and high-quality is defined as at least 7 stars.

### Statistical methods

All analyses were conducted by Stata version 11.0 (StateCorp, College Station, TX, USA). All statistical tests were two-sided, and a *P* < 0.05 was considered significant. The summary ORs were calculated by pooling RRs or ORs for highest *vs*. lowest categories of tea consumption from each study. The method designed by Greenland and Longnecker [51] and Orsini [52] (generalized least squares for trend estimation of summarized dose-response data) was performed for the dose-response analysis, requiring the doses of tea consumption, distributions of cases and person-years, RRs or ORs with 95% CIs for at least three quantitative exposure levels . As for different estimation criterions in each study, including cups of tea drinking, grams of tea leaves and frequency of tea drinking, we normalized the different exposure categories as 1 cup/day to calculate a risk estimate for an increment of tea consumption. To improve the accuracy of dose-response meta-analysis, we redefined tea consumption to the number of drinking cups per day, considering that 1 cup/day approximately equals to 150 g of tea leaves per month [53], 1 times/day, or daily drinker. Furthermore, if a study only supplied a range of exposure category, the midpoint of tea consumption was calculated as exposure level; if the highest category was open-ended, 1.2 times of the lowest bound was assumed as the dose [54]. The possible heterogeneity was assessed with χ^2^ test and *I*^2^ statistics, and the *I*^2^ statistic from 0 to 30% was considered as no or marginal heterogeneity, 30–75% as moderate heterogeneity and over 75% as significant heterogeneity [55]. The fixed effect model was performed only when there existed no significant heterogeneity; otherwise, the random effect model was used [56]. We further explored the heterogeneity through meta-regressions and stratified analysis. The potential confounders were as follows: study design (cohort, case-control, population based, hospital based), geographic area (Asia, America, Europe), tea source (green tea, any tea), gender (male, female), cancer location (colon, rectal, colorectal cancer), publication year (before 2000, year 2000 or later) and quality score (< 7 stars, ≥ 7 stars). The between-study variance (τ^2^) and its percentage were used to measure the heterogeneity degree and the extent of the explained heterogeneity of the characteristics, respectively. we removed one study once a time and conducted a sensitive analysis for the rest. Begg's funnel plots and the Egger's test were used to evaluate the publication bias with the significant set to *P* < 0.10 [57, 58].

### SUPPLEMENTARY MATERIALS FIGURES





## Abbreviations

OR: odds ratio
CIs: confidence intervals
CRC: colorectal cancer
EGCG: epigallcatechin-3-gallate
HCC: hospital-based case-control study
PCC: population-based case-control study
M: male
F: female.
