# Supplementary material for: An inverse association between tea consumption and colorectal cancer risk
Source: Oncotarget. 2017 Apr 8;8(23):37367–76. doi: 10.18632/oncotarget.16959 (PMC5514915; doi:10.18632/oncotarget.16959)
Supplement: Supplementary file 1 [file oncotarget-08-37367-s001.pdf]

An inverse association between tea consumption and colorectal cancer risk

Supplementary Materials

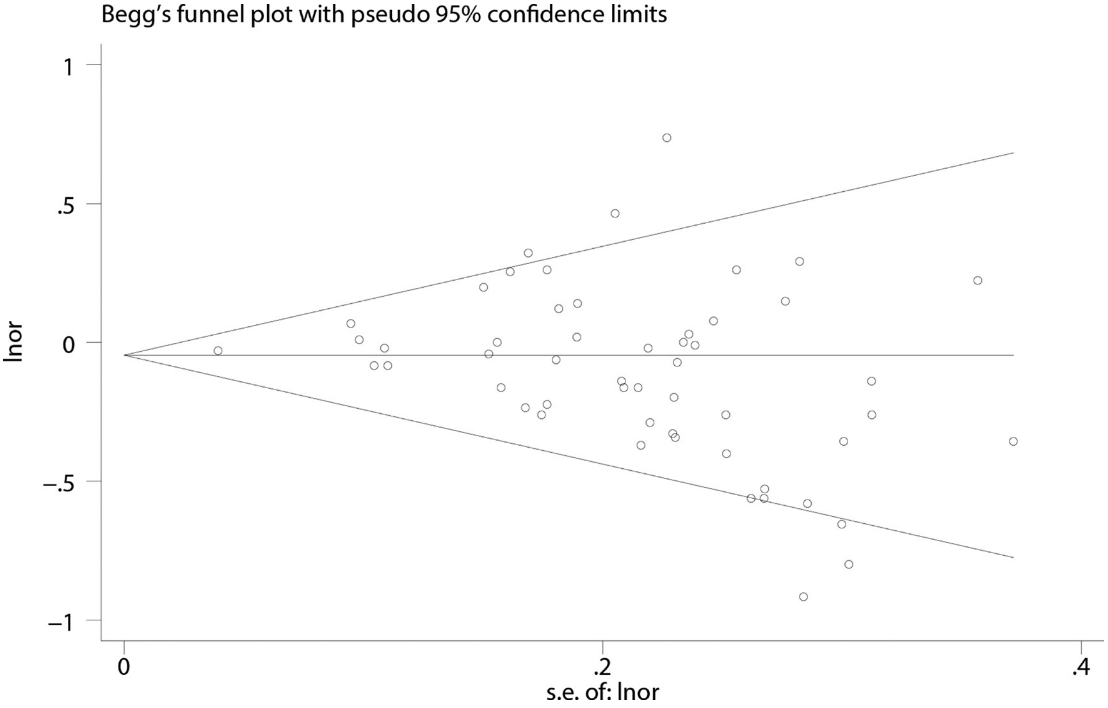

Supplementary Figure 1: Publication bias in the studies on highest vs. lowest level of tea consumption and colorectal cancer risk with Egger test.

Supplementary Table 1: Characteristics of cohort and case-control studies of tea consumption and colorectal cancer risk. See Supplementary_Table_1

Supplementary Table 2: Methodological quality of cohort studies included in the meta-analysis

Studies	Representativeness of the exposed cohort	Selection of the nonexposed cohort	Ascertainment of exposure	Outcome of interest not present at start of study	Control for important factors[a]	Assessment of outcome	Follow-up period long enough for outcomes to occur[b]	Adequacy of follow-up evaluation of cohorts[c]	Total quality scores
Goldbohm et al. 1996	☆	☆	☆	☆	☆	☆	-	☆	7
Zheng et al. 1996	☆	☆	☆	☆	-	☆	☆	-	6
Hartman et al. 1998	☆	☆	☆	☆	-	☆	☆	-	6
Nagano et al. 2001	☆	☆	☆	☆	-	☆	☆	-	6
Terry et al. 2001	☆	☆	☆	☆	☆	☆	☆	☆	8
Su et al. 2002	☆	☆	☆	☆	-	☆	☆	☆	7
Michels et al. 2005	☆	☆	☆	☆	-	☆	☆	☆	7
Suzuki et al. 2005	☆	☆	☆	☆	☆	☆	☆	☆	8
Oba et al. 2006	☆	☆	☆	☆	☆	☆	☆	☆	8
Lee et al. 2007	☆	☆	☆	☆	☆	☆	☆	-	7
Sun et al. 2007	☆	☆	☆	☆	☆	☆	☆	☆	8
Yang et al. 2007	☆	☆	☆	☆	-	☆	-	☆	6
Simons et al. 2010	☆	☆	☆	☆	-	☆	☆	-	6
Yang et al. 2011	☆	☆	☆	☆	-	☆	-	-	5
Nechuta et al. 2012	☆	☆	☆	☆	-	☆	-	☆	6
Sinha et al. 2012	☆	☆	☆	☆	-	☆	☆	☆	7
Dominianni et al. 2013	☆	☆	☆	☆	-	☆	☆	-	6

[a]This part has 2 stars at most. Studies that adjusted for age or coffee consumption deserved 1 star respectively.
[b]None star means a cohort study did not provide follow-up years. 1 star means a follow-up year over 5 in a cohort study.
[c]None star means a cohort study with no clear follow-up rate. 1 star means a follow-up rate over 80% in a cohort study.

Supplementary Table 3: Methodological quality of case-control studies included in the meta-analysis

Study	Adequate definition of cases	Representativeness of cases	Selection of controls	Definition of controls	Control for important factors[a]	Exposure ascertainment[b]	Same method of ascertainment for all subjects	Nonresponse rate[c]	Total quality rate
Baron et al. 1994	☆	☆	☆	☆	☆	-	☆	-	6
Ji et al. 1997	☆	☆	☆	☆	☆	-	☆	-	6
Munoz et al. 1998	☆	☆	☆	☆	☆	-	☆	☆	7
Tajima et al. 1998	☆	☆	☆	-	☆	-	☆	-	5
Slattery et al. 1999	☆	☆	☆	-	☆	-	☆	-	5
Cerhan et al. 2011	☆	☆	☆	☆	☆☆	-	☆	☆	8
Iiyasova et al. 2002	☆	☆	☆	-	☆	-	☆	-	5
Woolcott et al. 2002	☆	☆	☆	☆	☆	-	☆	☆	7
Iiyasova et al. 2003	☆	☆	☆	☆	☆	-	☆	☆	7
Li et al. 2011	☆	☆	☆	☆	☆	-	☆	☆	7
Wang et.al. 2013	☆	☆	☆	☆	☆	☆	☆	☆	8
Green et.al. 2013	☆	☆	☆	☆	☆	☆	☆	☆	8

[a]This part has 2 stars at most. Studies that adjusted for age or coffee consumption deserved 1 star respectively.
[b]2 stars could be given for this part at most. If studies did not provide the evidence about double-blind between case and control status or consideration about tea consumption changes because of disease, none star will be awarded.
[C]One star means that there was no significant difference in the response rate between case and control status by using the chi-square test ($P > 0.05$)

Supplementary Table 4: Meta-regression analysis

Variable	Coefficient	Standard error	*P* value	95% CI	
Publish year	−.0083075	.104799	0.937	−.2226454	.2060304
Region	.0644497	.043658	0.151	−.0248409	.1537403
Design	.0229578	.0538925	0.673	−.0872648	.1331804
Sex	−.0774416	.0799168	0.341	−.2408898	.0860066
Source	−.0825571	.1201193	0.497	−.3282287	.1631144
