# Supplementary material for: An inverse association between tea consumption and colorectal cancer risk
Source: Oncotarget. 2017 Apr 8;8(23):37367–76. doi: 10.18632/oncotarget.16959 (PMC5514915; doi:10.18632/oncotarget.16959)
Supplement: Supplementary file 2 [file oncotarget-08-37367-s002.docx]

Supplementary Table 1: Characteristics of cohort and case-control studies of tea consumption and colorectal cancer risk

| Study | Year | Design method | Study period | Population | Tea source | Lowest consumption level | Highest consumption level | Cancer site | Gender | OR/RR (95% CI) for highest versus lowest level | Adjustment factors |
| --- | --- | --- | --- | --- | --- | --- | --- | --- | --- | --- | --- |
| Cohort studies | |  |  |  |  |  |  |  |  |  |  |
| Goldbohm et.al | 1996 | Cohort | 1986-1991 | Netherlands | Black tea | Non-drinkers | ≥5 cups/day | Colorectum | All | 0.94(0.66-1.34) | Family history, body mass index, gallbladder surgery, and intake of fiber, folate, alcohol, and coffee, education, smoking status, and intakes of vitamin C. |
|  |  |  |  |  |  |  |  | Colon | Male | 1.01(0.53-1.91) |  |
|  |  |  |  |  |  |  |  |  | Female | 0.69(0.37-1.29) |  |
|  |  |  |  |  |  |  |  | Rectum | Male | 1.49(0.78-2.85) |  |
|  |  |  |  |  |  |  |  |  | Female | 0.71(0.29-1.72) |  |
| Zheng et.al | 1996 | Cohort | 1986-1993 | America | Tea(unclear) | Non-drinkers | ≥2 cups/day | Colon | Female | 0.71(0.45-1.11) | Age, education, smoking status, pack-years of smoking, physical activity, fruit and vegetable intake, and family history. |
|  |  |  |  |  |  |  |  | Rectum | Female | 0.70(0.34-1.46) |  |
| Hartman et.al | 1998 | Cohort | 1985-1993 | Finland | Tea(unclear) | Non-drinkers | ≥1 cup/day | Colon | Male | 2.09(1.34-3.26) | Age, intervention group, calcium, occupational physical activity, and BMI. |
|  |  |  |  |  |  |  |  | Rectum | Male | 0.87(0.47-1.60) |  |
| Terry et.al | 2001 | Cohort | 1987-1994 | Sweden | Tea(unclear) | <1 cup/week | ≥2 cups/day | Colorectum | Female | 0.98(0.64-1.51) | Age, body mass index, education level, quartiles of total calories, red meat, coffee, alcohol, energy-adjusted. |
|  |  |  |  |  |  |  |  | Colon | Female | 0.74(0.42-1.31) |  |
|  |  |  |  |  |  |  |  | Rectum | Female | 1.53(0.77-3.03) |  |
| Nagano et.al | 2001 | Cohort | 1979-1994 | Japan | Green tea | 0-1 cup/day | >5 cups/day | Colon | All | 1.00(0.76-1.40) | City, age, gender, radiation exposure, smoking status, alcohol drinking, body-mass index, education level, and calendar time. |
|  |  |  |  |  |  |  |  | Rectum | All | 1.30(0.77-2.10) |  |

Supplementary Table 1: Continued.

| Study | Year | Design method | Study period | Population | Tea source | Lowest consumption level | Highest consumption level | Cancer site | Gender | OR/RR (95% CI) for highest versus lowest level | Adjustment factors |
| --- | --- | --- | --- | --- | --- | --- | --- | --- | --- | --- | --- |
| Su et.al | 2002 | Cohort-1 | 1971-1975 | America | Tea(unclear) | Non-drinkers | >1.5 cups/day | Colon | All | 0.85(0.56-1.30) | Age, race, education level, BMI, aspirin use, dietary intakes of calories, fat, fiber and calcium, and alcohol. |
|  |  |  |  |  |  |  |  |  | Male | 0.53(0.26-1.11) |  |
|  |  |  |  |  |  |  |  |  | Female | 1.19(0.70-2.03) |  |
|  |  | Cohort-2 | 1971-1975 | America | Tea(unclear) | Non-drinkers | >1.5 cups/day | Colon | All | 0.59(0.35-1.00) |  |
|  |  |  |  |  |  |  |  |  | Male | 0.30(0.09-0.98) |  |
|  |  |  |  |  |  |  |  |  | Female | 0.74(0.40-1.39) |  |
| Michels et.al | 2005 | Cohort | 1980-1994 | America | Tea(unclear) | Non-drinkers | ≥2 cups/day | Colorectum | Female | 0.96(0.76-1.22) | Age, family history, history of sigmoidoscopy, height, body mass index, pack-years of smoking, physical activity, aspirin use, vitamin supplement intake, alcohol consumption, red meat consumption, total caloric intake, and, among women in addition for menopausal status, postmenopausal, hormone use. |
|  |  |  |  |  |  |  |  |  | Male | 1.12(0.78-1.59) |  |
|  |  |  |  |  |  |  |  |  | All | 1.01(0.83-1.22) |  |
|  |  |  |  |  |  |  |  | Colon | Female | 1.08(0.83-1.39) |  |
|  |  |  |  |  |  |  |  |  | Male | 1.05(0.69-1.59) |  |
|  |  |  |  |  |  |  |  |  | All | 1.07(0.86-1.33) |  |
|  |  |  |  |  |  |  |  | Rectum | Female | 0.54(0.29-0.99) |  |
|  |  |  |  |  |  |  |  |  | Male | 1.34(0.67-2.68) |  |
| Suzuki et.al | 2005 | Cohort-1 | 1984-1992 | Japan | Green tea | <1 cup/day | >5 cups/day | Colon | All | 1.03(0.65-1.64) | Sex, age, family history of colorectal cancer, cigarette, alcohol consumption, body mass index, consumption of black tea and coffee, consumption of meat, green-yellow vegetables, other vegetables, and fruits. |
|  |  | Cohort-2 | 1990-1997 | Japan | Green tea | <1 cup/day | >5 cups/day | Colon | All | 0.93(0.59-1.46) |  |
|  |  | Cohort1+2 |  |  |  |  |  |  | Male | 1.12(0.72-1.74) |  |
|  |  | Cohort1+2 |  |  |  |  |  |  | Female | 0.79(0.49-1.29) |  |
|  |  | Cohort-1 | 1984-1992 | Japan | Green tea | <1 cup/day | >5 cups/day | Rectum | All | 1.34(0.77-2.33) |  |
|  |  | Cohort-2 | 1990-1997 | Japan | Green tea | <1 cup/day | >5 cups/day | Rectum | All | 0.57(0.34-0.95) |  |
|  |  | Cohort1+2 |  |  |  |  |  |  | Male | 0.62(0.38-1.02) |  |
|  |  | Cohort1+2 |  |  |  |  |  |  | Female | 1.30(0.70-2.42) |  |

Supplementary Table 1: Continued.

| Study | Year | Design method | Study period | Population | Tea source | Lowest consumption level | Highest consumption level | Cancer site | Gender | OR/RR (95% CI) for highest versus lowest level | Adjustment factors |
| --- | --- | --- | --- | --- | --- | --- | --- | --- | --- | --- | --- |
| Oba et.al | 2006 | Cohort | 1992-2000 | Japan | Tea(unclear) | never to <1 cup/month | >1 cup/day | Colon | Male | 0.75(0.49-1.16) | Age, height, BMI, total pack-years of cigarette smoking, alcohol intake, physical activity, black tea intake and green tea/coffee intake. |
|  |  |  |  |  |  |  |  |  | Female | 1.08(0.67-1.76) |  |
| Sun et.al | 2007 | Cohort | 1993-2004 | Singapore | Green tea | Non-drinker | Daily | Colorectum | All | 1.18(0.97-1.45) | Sex, age, year of interview, dialect group, education, family history, history of diabetes, cigarette smoking, alcohol drinking, coffee drinking, weekly moderate physical activity, body mass index, total energy, total fat, dietary fiber, calcium, vitamin, black tea intake, and green tea intake. |
|  |  |  |  |  | Black tea | Non-drinker | Daily |  | All | 0.92(0.73-1.16) |  |
|  |  |  |  |  | Any tea | Non-drinker | Daily |  | All | 1.07(0.89-1.29) |  |
|  |  |  |  |  | Green tea | Non-drinker | Daily | Colorectum | Male | 1.36(1.06-1.74) |  |
|  |  |  |  |  | Black tea | Non-drinker | Daily |  | Male | 0.87(0.66-1.15) |  |
|  |  |  |  |  | Any tea | Non-drinker | Daily |  | Male | 1.18(0.93-1.50) |  |
|  |  |  |  |  | Green tea | Non-drinker | Daily | Colorectum | Female | 0.91(0.63-1.32) |  |
|  |  |  |  |  | Black tea | Non-drinker | Daily |  | Female | 1.03(0.67-1.57) |  |
|  |  |  |  |  | Any tea | Non-drinker | Daily |  | Female | 0.92(0.67-1.25) |  |
| Yang et.al | 2007 | Cohort | 1996-2004 | Shanghai | Green tea | Non-drinker | ≥5g/day | Colorectum | Female | 0.56(0.32-0.98) | Age, education, household income, cigarette smoking, alcohol drinking, physical activity, body mass index, menopausal status, drug use, vitamin supplement use, prior histories of colorectal polyps and chronic ulcerative colitis, family history, and intakes of total energy, vegetables, fruits, and red meat. |

Supplementary Table 1: Continued.

| Study | Year | Design method | Study period | Population | Tea source | Lowest consumption level | Highest consumption level | Cancer site | Gender | OR/RR (95% CI) for highest versus lowest level | Adjustment factors |
| --- | --- | --- | --- | --- | --- | --- | --- | --- | --- | --- | --- |
| Lee et.al | 2007 | Cohort | 1990-2002 | Japan | Green tea | Almost never | >5 cups/day | Colorectum | Male | 0.96(0.71-1.29) | Age and study area, BMI, smoking status, alcohol drinking, family history, physical activity, and intake of green, vegetables, beef, pork, coffee, Chinese tea and black tea. |
|  |  |  |  |  |  |  |  |  | Female | 1.02(0.70-1.47) |  |
|  |  |  |  |  |  |  |  | Colon | Male | 0.92(0.63-1.33) |  |
|  |  |  |  |  |  |  |  |  | Female | 1.10(0.70-1.73) |  |
|  |  |  |  |  |  |  |  | Rectum | Male | 1.04(0.63-1.72) |  |
|  |  |  |  |  |  |  |  |  | Female | 0.85(0.45-1.61) |  |
| Simons et.al | 2010 | Cohort | 1986-1999 | Netherlands | Tea(unclear) | ≤1 cup/day | >3 cups/day | Colorectum | Male | 0.92(0.75-1.13) | Age, family history, physical activity, smoking status, educational level, body mass index, ethanol intake, meat intake, processed meat intake, folate intake, vitamin B6 intake, fiber intake, and fluid intake from other fluids |
|  |  |  |  |  |  |  |  |  | Female | 0.92(0.74-1.14) |  |
|  |  |  |  |  |  |  |  | Rectum | Male | 0.85(0.63-1.16) |  |
|  |  |  |  |  |  |  |  |  | Female | 1.00(0.66-1.51) |  |
| Yang et.al | 2011 | Cohort | 2002-2008 | Shanghai | Green tea | Non-drinker | ≥250 g/month | Colorectum | Male | 0.85(0.62-1.15) | Age, education, cigarette smoking, pack-years of cigarette smoking, alcohol consumption, regular exercise, Body Mass Index, history of diabetes, family history and intakes of vegetables, fruits and red meat. |
|  |  |  |  |  |  |  |  | Colon | Male | 0.69(0.48-0.98) |  |
|  |  |  |  |  |  |  |  | Rectum | Male | 0.89(0.59-1.34) |  |
| Nechuta et.al | 2012 | Cohort | 1996-2009 | Shanghai | Tea(unclear) | Non-drinker | ≥150 g/month | Colorectum | Female | 0.86(0.63,1.18) | Age, marital status, education, occupation, BMI, exercise, fruit and vegetable intake, meat intake, diabetes, and family history. |
|  |  |  |  |  |  |  |  | Colon | Female | 0.85(0.56,1.27) |  |
|  |  |  |  |  |  |  |  | Rectum | Female | 0.89(0.55,1.43) |  |

Supplementary Table 1: Continued.

| Study | Year | Design method | Study period | Population | Tea source | Lowest consumption level | Highest consumption level | Cancer site | Gender | OR/RR (95% CI) for highest versus lowest level | Adjustment factors |
| --- | --- | --- | --- | --- | --- | --- | --- | --- | --- | --- | --- |
| Sinha et.al | 2012 | Cohort | 1995-1996 | America | Tea(unclear) | Non-drinker | ≥1 cup/d | Colorectum | All | 0.97(0.90,1.05) | Age, sex, race, education, smoking status, time since quitting for former smokers, smoking dose, diabetes, colorectal screening family history of colorectal cancer, regular drug use, marital status, BMI, frequency of vigorous physical activity, calories, fruit and vegetables, red meat, dietary calcium intake, alcohol, and menopausal hormone therapy in women. |
|  |  |  |  |  |  |  |  | Colon | All | 0.99(0.91,1.08) |  |
|  |  |  |  |  |  |  |  | Rectum | All | 0.92(0.80,1.07) |  |
| Dominianni et.al | 2013 | Cohort | 1993-2001 | America | Tea(unclear) | Non-drinker | ≥2 cups/d | Colorectum | All | 0.77(0.55-1.09) | Age, gender, race, family history of colorectal cancer, education, body mass index, physical activity, smoking status, NSAID intake, history of diabetes, number of colorectal examinations up to 3 years before the start of study, hormone use, fruit intake, vegetable intake, meat intake, alcohol intake and study centre. |
|  |  |  |  |  |  |  |  | Rectum | All | 0.70(0.33-1.46) |  |

Supplementary Table 1: Continued.

| Study | Year | Design method | | Study period | | Population | | | Tea source | Lowest consumption level | | Highest consumption level | | Cancer site | | Gender | | OR/RR (95% CI) for highest versus lowest level | | Adjustment factors |
| --- | --- | --- | --- | --- | --- | --- | --- | --- | --- | --- | --- | --- | --- | --- | --- | --- | --- | --- | --- | --- |
| Population-based case-control (PCC) studies | | | | | | | |  | | |  | |  | |  | |  | |  |  |
| Baron et.al | 1994 | | PCC | | 1986-1988 | | Sweden | Tea(unclear) | | | Non-drinker | | >2 cups/day | | Colon | | All | | 0.96(0.67-1.37) | intake of fat and fiber, BMI, and exercise. |
|  |  | |  | |  | |  |  | | |  | |  | | Rectum | | All | | 0.56(0.34-0.90) |  |
|  |  | |  | |  | |  |  | | |  | |  | | Colorectum | | All | | 0.79(0.57-1.10) |  |
| Ji et.al | 1997 | | PCC | | 1990-1993 | | Shanghai | Green tea | | | Non-drinker | | ≥300 g/month | | Colon | | Male | | 0.82(0.52-1.28) | Age, income, education and cigarette smoking. |
|  |  | |  | |  | |  |  | | |  | |  | | Rectum | | Female | | 0.72(0.46-1.13) |  |
|  |  | |  | |  | |  |  | | |  | |  | | Colon | | Male | | 0.67(0.41-1.10) |  |
|  |  | |  | |  | |  |  | | |  | |  | | Rectum | | Female | | 0.57(0.34-0.97) |  |
| Slattery et.al | 1999 | | PCC | | 1991-1994 | | America | Caffeinated tea | | | Non-drinker | | >1 serving/day | | Colon | | All | | 0.98(0.79-1.21) | Age, BMI, long-term vigorous leisure-time physical activity, total energy intake, sucrose, usual number of cigarettes smoked and alcohol. |
|  |  | |  | |  | |  | Herbal tea | | | Non-drinker | | >1 serving/day | | Colon | | All | | 0.87(0.58-1.31) |  |
| Woolcott et.al | 2002 | | PCC | | 1992-1994 | | Canada | Tea(unclear) | | | <1 cup/day | | ≥5 cups/day | | Colon | | All | | 1.13(0.79-1.61) | Age, sex, education level, body mass index and intake of energy, calcium, fiber and cholesterol. |
|  |  | |  | |  | |  |  | | |  | |  | |  | | Female | | 1.44(0.90-2.29) |  |
|  |  | |  | |  | |  |  | | |  | |  | |  | | Male | | 0.73(0.41-1.31) |  |
|  |  | |  | |  | |  |  | | |  | |  | | Rectum | | All | | 1.15(0.79-1.66) |  |
|  |  | |  | |  | |  |  | | |  | |  | |  | | Female | | 1.30(0.78-2.17) |  |
|  |  | |  | |  | |  |  | | |  | |  | |  | | Male | | 0.95(0.55-1.64) |  |
| Ilyasova et.al | 2003 | | PCC | | 1998-1999 | | Moscow | Tea(unclear) | | | <80g/month | | >160g/month | | Rectum | | Female | | 0.40(0.23-0.70) | Age, BMI, alcohol, and exposure to fried meat. |
|  |  | |  | |  | |  |  | | |  | |  | |  | | Male | | 0.77(0.42-1.43) |  |
| Ilyasova et.al | 2003 | | PCC | | 1996-2000 | | America | Tea(unclear) | | | Non-drinker | | ≥2servings/day | | Colon | | All | | 1.30(0.90-1.80) | Age, gender, and race. |

Supplementary Table 1: Continued.

| Study | Year | Design method | Study period | Population | Tea source | Lowest consumption level | Highest consumption level | Cancer site | Gender | OR/RR (95% CI) for highest versus lowest level | Adjustment factors |
| --- | --- | --- | --- | --- | --- | --- | --- | --- | --- | --- | --- |
| Li et.al | 2011 | PCC | 2009-2010 | Jiangsu | Green tea | Non-drinker | ≥1 times/day | Colon | All | 0.62(0.42-0.92) | Terms for education, BMI (5 years ago), smoking, passive smoking, alcohol consumption, physical activity, energy intake, cancer in first degree relative. |
|  |  |  |  |  |  |  |  |  |  |  |  |
| Cerhan et.al | 2011 | PCC | 1986-1989 | America | Hot tea | Non-drinker | >3 cups/day | Colon | All | 1.40(0.80-2.50) | Age, sex, education, smoking history, leisure physical activity, and intake of dietary fiber and fruits and vegetables. |
|  |  |  |  |  | Iced tea | Non-drinker | >5 cups/day |  | All | 0.50(0.20-1.10) |  |
|  |  |  |  |  | Total tea | Non-drinker | >5 cups/day |  | All | 0.70(0.40-1.30) |  |
|  |  |  |  |  | Hot tea | Non-drinker | >3 cups/day | Rectum | All | 1.10(0.60-1.90) |  |
|  |  |  |  |  | Iced tea | Non-drinker | >5 cups/day |  | All | 1.00(0.50-1.80) |  |
|  |  |  |  |  | Total tea | Non-drinker | >5 cups/day |  | All | 1.00(0.60-1.50) |  |
| Wang et.al | 2013 | PCC | 2000-2003 | Japan | Tea polyhenols | 65mg/d(median) | 853mg/d(median) | colorectum | all | 1.38(0.99-1.92) | Adjusted for age, sex, residence area, parental history of colorectal cancer, smoking, alcohol drinking, body mass index 10 years before, type of job , leisure-time physical activity,calcium and n-3 polyunsaturated fatty acids. |
| Green et.al | 2013 | PCC | 2005-2007 | Australia | Back tea | Non-drinker | ≥1 cup/day | Rectum | All | 1.31(0.85-2.04) | Adjusted for age group, sex, energy intake from food, alcohol intake, smoking status, use of multivitamins, diabetes, physical activity during the age period 19–34 years, body mass index at age 40 years, socioeconomic status, and country of birth. |
|  |  |  |  |  | Tea with milk | Non-drinker | ≥2 cup/day |  | All | 1.30(0.86-1.95) |  |
|  |  |  |  |  | Green tea | Non-drinker | ≥1 cup/day |  | All | 1.05(0.53-2.10) |  |
|  |  |  |  |  | Herbal tea | Non-drinker | ≥1 cup/day |  | All | 0.73(0.39-1.35) |  |
| Hospital-based case-control (HCC) studies | | | | | |  |  |  |  |  |  |
| Munoz et.al | 1998 | HCC | 1993-1997 | Argentina | Tea(unclear) | Non-drinker | ≥1 cup/day | Colon | All | 0.80(0.60-1.20) | Age, sex, social class, and body mass index. |
| Inoue et.al | 1998 | HCC | 1990-1995 | Japan | Green tea | Non-drinker | >7 cups/day | Colon | All | 0.77(0.47-1.26) | Green tea intake, coffee intake, black tea intake, gender, age, year and season at first hospital-visit, habitual smoking, habitual alcohol drinking, regular physical exercise, fruit intake, rice intake, and beef intake |
|  |  |  |  |  |  |  |  | Rectum | All | 1.25(0.62-2.51) |  |
|  |  |  |  |  | Black tea | Non-drinker | ≥1 cup/day | Colon | All | 1.59(1.06-2.37) |  |
|  |  |  |  |  |  |  |  | Rectum | All | 1.16(0.67-0.45) |  |
| Li et.al | 2011 | HCC | 2009-2010 | Jiangsu | Green tea | Non-drinker | ≥1 times/day | Colon | All | 0.67(0.45-0.99) | Terms for education, BMI, smoking, passive smoking, alcohol consumption, physical activity, energy intake, cancer in first degree relative. |
